# Does Engaging in a Group-Based Intervention Increase Parental Self-efficacy in Parents of Preschool Children? A Systematic Review of the Current Literature

**DOI:** 10.1007/s10826-016-0464-z

**Published:** 2016-06-13

**Authors:** Anja Wittkowski, Hannah Dowling, Debbie M. Smith

**Affiliations:** School of Psychological Sciences, 2nd Floor Zochonis Building, Brunswick Street, Manchester, M13 9PL UK

**Keywords:** Parental, Self-efficacy, Preschool children, RCT, Parenting intervention

## Abstract

As the preschool years are a formative period for long-term physical and mental health, this period is recognised as an important window for early effective intervention. Parenting behaviour is a key factor to target in order to optimise child development. Group-based interventions for parents are considered efficient and cost effective methods of early intervention and have been found to improve child behaviour and adjustment. Self-efficacy is key to behaviour change and as such parental self-efficacy should be a consideration in interventions aimed at influencing parenting behaviour. Therefore, the purpose of this systematic review was to examine the impact of group-based early interventions for parents of preschool children on parental self-efficacy. Nine databases were searched (ASSIA, CINAHL, EMBASE, Maternity and Infant Care, Ovid Medline, PsycINFO, Pubmed, Science Direct and Web of Science). Studies were included if they were a randomised controlled trial of a group-based intervention for parents of preschool children and measured change in parental self-efficacy. Fifteen studies were identified. Although changes in parental self-efficacy following a group-based intervention were noted in the majority of studies reviewed, the methodological quality of the studies included in the review means these findings have to be interpreted with caution; only seven studies were rated to be methodologically adequate. Further research is needed to understand the mechanisms by which these interventions may improve parental self-efficacy. Studies specifically examining the impact of such interventions on paternal self-efficacy are also warranted.

## Introduction

The preschool years, defined in this review as 0–6 years of age in line with international school starting ages (Sargent et al. 2013), are a period of rapid physical and psychological development for children. The need to implement early intervention strategies to give children the best opportunity to strive and pre-empt later personal and societal level problems has been widely recognised (e.g. Allen 2011). To this end, the UK Government emphasised the need for evidence-based preventative interventions aimed at increasing the parental competence of parents of preschool children (Allen 2011; Leadsom et al. 2014).

Parents are integral in shaping their child’s physical, emotional and social environment and thus their development. According to Waldfogel and Washbrook (2011), parental behaviours play a significant role in a child’s psychosocial development even after controlling for demographic characteristics. Lack of positive attention from parents paired with inconsistent and inappropriate discipline has been found to be predictive of anti-social behaviour, conduct disorder and criminality in later life (Farrington 2005). Conversely, positive reinforcement, responsiveness, warmth and positive affect have been associated with positive child developmental outcomes (Gardner et al. 2003; Shaw et al. 1998; Zhou et al. 2008). As parenting style has been shown to be adaptable to change (Taylor and Biglan 1998; Wykes et al. 2008), a pivotal mechanism for early intervention is through parents and promoting effective parenting.

According to Bandura (1982, 1997), self-efficacy has been defined as an individual’s beliefs about their capabilities to produce desired levels of performance to influence events that affect their lives (Bandura 1982). Thus, self-efficacy is central to conducting behaviour and it influences behaviour change. Parental self-efficacy is a subcategory of general self-efficacy and has been broadly defined as the expectation a parent holds about their ability to parent successfully (Jones and Prinz 2005). Strong evidence for a link between parental self-efficacy and parental competence has been found, with some evidence that higher levels of parental self-efficacy are related to more effective parenting and better child outcomes (see Jones and Prinz 2005 for a review). In addition, high maternal self-efficacy has been linked to increased sensitivity and responsiveness towards their child in longitudinal studies (Dumka et al. 2010; Teti and Gelfand 1991) as well as to increased maternal warmth in cross-sectional studies (Izzo and Weiss 2000). These factors in turn have been found to predict decreases in child aggression (Jones et al. 2008) and positive social-emotional development in children in longitudinal studies (Page et al. 2010). In contrast, a link has been found between lower parental self-efficacy and higher dysfunctional parenting, including laxness and over-reactivity, in several cross-sectional studies (e.g., Gross et al. 1999; Sanders and Woolley 2005).

As self-efficacy is not a fixed personality trait but a dynamic process modified by task and situational demands as well as changing individual factors (Bandura 1997), parental self-efficacy should be a crucial consideration when assessing parenting interventions aimed at increasing parental competence. Support has been found for multiple mechanisms through which parental self-efficacy influences parental behaviour, indicating parental self-efficacy can be an antecedent, consequence and mediator of parenting.

High parental self-efficacy has been linked to positive parenting strategies and behaviours (Coleman and Karraker 1998). When parents felt competent in their ability to parent, they were likely to use more effective parenting practices, which foster positive developmental outcomes (Bloomfield et al. 2005). According to Coleman and Karraker (1998), self-efficacy beliefs influence parenting behaviours via a dynamic interaction of affective, motivational, cognitive and behavioural pathways. Low self-efficacy can also have a direct impact on behaviour through inhibiting the acquisition of new skills and suppressing existing skills (Bandura 1982), which is particularly pertinent when considering how to optimise the acquisition and use of positive parenting skills. Effective parenting leads to enhanced feelings of efficacy in a parent (Bandura 1997). A number of factors influence parental self-efficacy, including social support, infant temperament and maternal mental health (Cutrona and Troutman 1986; Leahy-Warren and McCarthy 2011; Leerkes and Burney 2007). Consequently, it is possible to identify particular groups of parents that are at risk of experiencing low parental self-efficacy. Parental self-efficacy may also mediate the effects of depression, perception of infant temperament and social support on parenting competence in parents of young children (Teti and Gelfand 1991). It has also been found to act as a buffer against the impact of adversity (e.g., Ardelt and Eccles 2001), which suggests that parental self-efficacy is a potential area of intervening and attenuating the effects of non-manipulatable variables, such as temperament. The evidence presented so far highlights a complicated relationship between parental self-efficacy and parenting behaviours, but nonetheless points to parental self-efficacy as an important factor to be targeted in parenting interventions.

Parental self-efficacy has been measured exclusively via self-report questionnaires and at different levels: general, task specific and narrow domain self-efficacy (Jones and Prinz 2005). General parental self-efficacy measures ask a parent to comment on how competent they feel in the parenting role without focussing on specific tasks. Task-specific measures calculate global parental self-efficacy by focusing on the perception of competence over a range of discrete parenting tasks; for example, discipline and soothing a baby. Narrow-domain measures concentrate on perceptions of competency in one parenting domain; for example, involvement in school-related activities. In a study assessing the relationship between maternal self-efficacy, dysfunctional discipline style and child conduct problems, Sanders and Woolley (2005) found differing results depending on the type of measure used and the domain assessed. Parenting practise was best predicted by scores on task-specific measures of parental self-efficacy.

Given the mediating role of self-efficacy between knowledge and behaviour (e.g., Bandura 1982, 1997), the impact of group-based programmes designed to impact on parenting skill on parental self-efficacy is an area of increased interest. Perceptions of self-efficacy also determine the amount of effort an individual expends and how long they persevere in the face of adversity (Bandura and Schunk 1981). Low self-efficacy can inhibit the acquisition of new skills and suppress existing skills (Bandura 1982). This is particularly relevant to parenting interventions: increasing skill and knowledge may only lead to behavioural change if a parent also has sufficient confidence in their abilities. Indeed, Grusec et al. (1994) noticed that parents with low parental self-efficacy were not able to put parenting knowledge into practice. Coleman and Karraker (1998) suggested that traditional parenting interventions focussing on knowledge and skills alone might not suffice. Thus, in order to optimise parenting quality it may be necessary to ensure parenting groups also increase parental self-efficacy. According to Sanders and Mazzucchelli (2013), parental self-efficacy is an important element of a broader capacity of self-regulation, important for nurturing positive parenting practises. They argued that integrating a focus on parental self-regulation into parenting interventions would allow parents to become self-sufficient in creating and maintaining change (see Sanders and Mazzucchelli 2013, for a full discussion).

Much of the research into the effectiveness of group-based interventions for parents has focussed on parenting programmes (see Barlow et al. 2012; Nowak and Heinrichs 2008, for reviews). Research has been conducted into groups based on a range of theoretical perspectives, including behavioural, cognitive-behavioural, psychodynamic and social learning theory. Despite differing in orientation, parenting programmes have been found to exhibit many commonalities in their implementation (Kazdin 1997). Often, although not always, sessions involve some elements of video vignettes, role-play or an opportunity to practise new techniques, interaction coaching, didactic teaching and group discussion to help parents develop the parent–child relationship elicit their own problem-solving skills, and provide an environment within which to practise these new skills.

Several previous reviews have found evidence that group-based parenting programmes were an effective intervention for reducing child problem behaviour (Dretzke et al. 2005; Taylor and Biglan 1998; Wyatt Kaminski et al. 2008), improving positive parenting, reducing harsh parenting practises, improving emotional and behaviour adjustment in children (Barlow et al. 2005, 2011) and improving parental psychosocial adjustment (Barlow et al. 2012). Additional benefits of group-based interventions include increased access to services and cost effectiveness, especially for children with challenging behaviour (Edwards et al. 2007; Scott et al. 2001). Five influential factors that construct and enhance self-efficacy have been identified: previous experience, vicarious experience, mastery experience, verbal (social) persuasion and psychological state (Bandura 1977, 1997). Group-based programmes are the most common form of parenting interventions and have the potential to offer a supportive environment to utilise these factors to influence parental self-efficacy. For example, verbal persuasion is more likely to occur in a social setting such as a group-based intervention. Most parenting-interventions are run in a group-based setting.

Parenting groups are not the only group-based programmes available to pre-school parents. Many structured group activities are available in the UK, such as music groups, swimming groups and sensory groups. These share many similar characteristics to parenting groups albeit the content is different. Similarities include being facilitated by an instructor, incorporating a taught element for parents and a focus on developing parent-infant relationships and parenting skills. Often participation in these group-based activities provides the parent and child with an instant opportunity to practise new skills, alongside didactic teaching, parental discussion and problem solving. Key concepts identified by Kane et al. (2007) as important in providing helpful and meaningful parenting programmes (e.g., the acquisition of knowledge, skills and understanding alongside social support) are also present in non-parenting programmes.

A Cochrane review examined the impact of group interventions on parental psychological functioning including depression, anxiety, general self-esteem and parental self-efficacy (Barlow et al. 2012). Overall, the review concluded that group-based parenting programmes significantly increased short-term psychological wellbeing in parents, including significant increases in parental self-efficacy. However, although parental self-efficacy was examined as one factor in this review, the impact of group-based interventions on parental self-efficacy specifically in parents of preschool children was not the focus of their review. Thus, the aim of this paper is to critically examine the literature on the impact of group-based interventions for parents of preschool children on parental self-efficacy and to assess the methodological qualities of each identified study. As the preschool years have been identified as a key period for intervention, we focussed on studies of parents of children up to 6-years old.

The quality of the evidence, the impact of the type of parental self-efficacy measure used and the role of potential moderating and mediating factors that influence whether group-based interventions impact on parental self-efficacy were also examined.

## Method

### Search Strategy

A systematic search of nine databases was conducted in January 2014 (ASSIA, CINAHL, EMBASE, Maternity and Infant Care, Ovid Medline, PsycINFO, Pubmed, Science Direct and Web of Science). All searches were restricted to randomised control trials written in English. No restriction was placed on the year of publication. The following keywords and their combinations using Boolean AND/OR operators were employed across all databases searched: Parent* OR Maternal OR Mother OR Father OR Paternal AND Child* OR Toddler OR Infan* OR bab* OR pre-schooler AND Therap* OR Intervention* OR group OR Support OR Activity OR program* OR education AND “Parent* confidence” OR “mater* confidence” OR “pater* confidence” OR “Parent* self-efficacy” OR “mater* self-efficacy” OR “pater* self-efficacy” OR “Parent* competence” OR “mater* competence” OR “pater* competence”.

### Inclusion Criteria

Studies were considered for inclusion if they utilised a randomised control trial design to evaluate a group intervention for parents of preschool children and assessed parental self-efficacy as an outcome measure. Relevant studies were identified by reference to assessing parental self-efficacy, parental self-confidence or parental competence. The type of group intervention offered and the length of intervention were not restricted because the aim was to identify the range of group interventions in which self-efficacy was assessed. Interventions which were mainly group-based but supplemented with individual sessions were included in the review. Although children enter the UK school system the school year they turn five, studies with parents of children up to the age of six were included to fit in with the international age for starting school (Sargent et al. 2013). The wide age range from birth to 6 years was chosen in line with the UK government’s focus on early years and preschool provision and in order to capture a wider range of group-based interventions parents take part in.

### Exclusion Criteria

Studies were excluded if the intervention was not in a group format, did not report parental self-efficacy as an outcome measure, if the type of self-efficacy measure could not be determined, and if the sample included parents of children aged over 6-years old. Studies written in a language other than English, reviews, book chapters and non-peer reviewed studies were also excluded.

### Study Selection

**Fig. 1:**
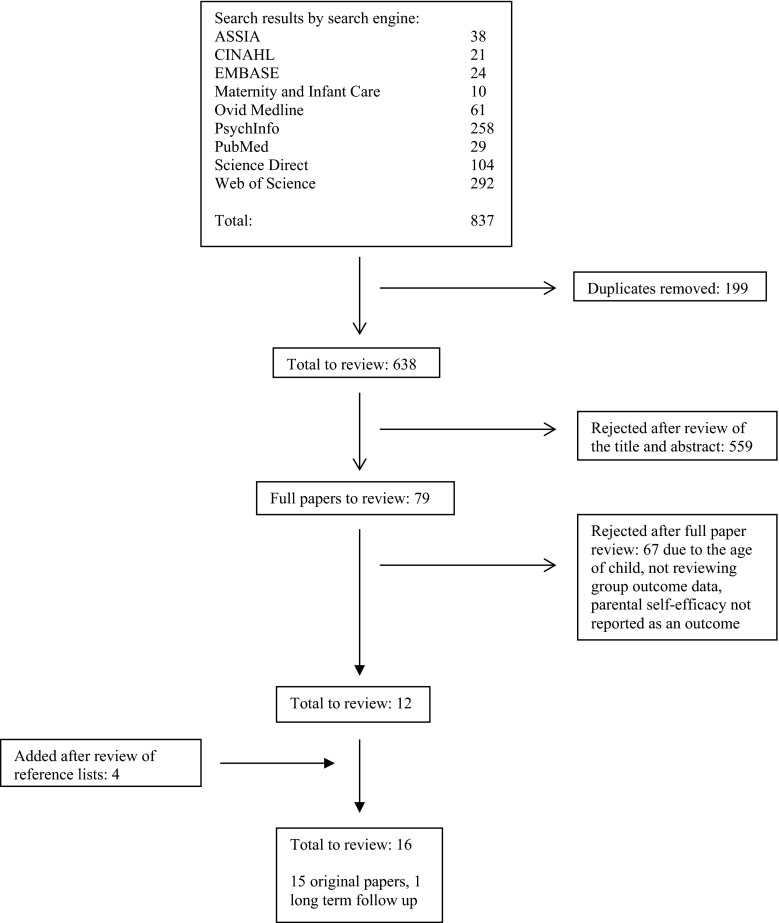
Schematic review of paper selection

Overall 837 studies were identified in the initial search, of which 199 duplicates were removed. Following a review of the title and abstract by one of the authors (HD), 559 did not meet inclusion criteria. A further 67 were excluded after full text examination (due to child age criterion not being met, no pre-post measures of change, parental self-efficacy not reported as an outcome measure and/or not a group intervention), leaving 12 studies for inclusion. An additional four were identified following an examination of reference lists, resulting in 16 studies to be considered for inclusion. As one paper (Tucker et al. 1998) presented the 1-year-follow up data of another included paper (Gross et al. 1995), we will refer to it only when discussing the follow up data for Gross et al. (1995). A total of 15 studies were therefore included in this literature review (see Fig. 1 for schematic diagram of paper selection).

### Quality Rating

The Clinical Tool for Assessment of Methodology (CTAM, Tarrier and Wykes 2004) was used to rate the methodological quality of the identified studies. The CTAM was chosen because it provides an overall representation of methodological rigour of randomised controlled trials. It consists of six subscales: sample size, allocation, assessment method, control group, analysis method and description of treatment the total of which provides an overall quality rating of the study. Scores range from 0 to 100. A total CTAM score of 65 or above represents adequate methodology (Wykes et al. 2008).

All studies were initially reviewed by one of the authors (HD) and all authors agreed with the findings. Twenty-five percent of the studies were reviewed by another researcher, experienced with the CTAM but independent to the research team, to check for consistency. The inter-rater correlation coefficient was found to be 0.97. Any discrepancies between the raters were discussed and a consensus reached following further examination of each study.

### Effect Sizes

Effect sizes were calculated using Cohen’s *d* to indicate the magnitude of difference of parental self-efficacy between the group intervention and no intervention control group at post-intervention in all studies that reported means and standard deviations. For Cohen’s *d,* effect sizes were considered small if they were between 0.2 and 0.3, medium if between 0.4 and 0.7 and large if over 0.8 (Cohen 1988).

## Results

### Overview of Studies

#### Location

The majority of the 15 studies were North American with seven studies from the USA and three studies from Canada. The remaining five studies were from Australia (see Table 1 for an overview of the reviewed studies).

**Table 1:** Overview of all included studies

Author, country and sample size	Participant characteristics	Inclusion criteria Exclusion criteria	Summary of intervention Control group	Number of sessions	Follow up?	Parental self-efficacy measure	Key findings	Effect sizes	Total CTAM score
*Studies utilising task*-*specific parental self*-*efficacy measure*									
Adamson et al. (2013) Australia 96 (94 mothers, 2 fathers)	Parents: intervention: 35.62 years, control 35.58 years Children: 1.5–6 years Average age: 3.14 years	Inclusion: Family concern about feeding and lived in areas where intervention was offered Exclusion: Families of children with medical conditions, those accessing other interventions, living outside the intervention area	Behavioural intervention; Triple P, Hassle free meal times Programme topics: strategies for successful mealtimes, promoting positive child behaviour, coping skills. Teaching methods: didactic instruction, active skills training, homework tasks. Waitlist control	4 × 2 h group sessions plus 3 telephone sessions.	6 month follow up for intervention group only	PTC	Greater behavioural parental self-efficacy reported by intervention group above control group; maintained at 6 month follow up. Clinically significantly change in PTC behaviour scores. No significant difference on PTC setting scale	PTC behaviour subscale 0.98 (large)	82
^a^Breitenstein et al. (2012) USA 504	Parents: Not stated Children: range not stated. Average age: 2.81 years	Inclusion: Latino or African American parents Exclusion: Not stated	Cognitive behavioural intervention; Chicago Parent Program (based on Incredible Years Program; adapted for African American and Latino parents) Programme topics: building parent–child relationships, child behavioural management, stress management, problem solving skills. Teaching methods: video vignettes to stimulate discussion and problem solving. Study 1: Waitlist control Study 2: TAU	12 × 2 h group sessions	6 and 12 month follow ups	TCQ	Significant improvement in parental self-efficacy in intervention group compared to control group post-intervention. Effects were maintained for intervention group, but no difference between groups at follow ups; indicating the control group also improved over time. Significant effect of race on results; Latino parents reported greater improvements in parental self-efficacy.	−0.01 (no effect)	74
Gross et al. (1995) USA 24 families	Mothers: 32 years Fathers 33 years Children: 24–36 months Average age: not stated	Inclusion: Child aged between 24 and 36 months. Both parents willing to participate in program. Questionnaires completed at pre, post and follow up. Child scores greater than 125 on intensity scale of ECBI or greater than 10 on the problem scale. Exclusion: Not stated	Cognitive behavioural intervention; Webster-Stratton Incredible Years Programme topics: how to play with a child, use of praise and rewards, limit setting, and managing misbehaviour. Teaching methods: Video-tape vignettes, group discussion and problem solving, homework tasks. Control and dropout group	10 × 2 h weekly sessions.	1 year follow up (Tucker et al. 1998 paper)	TCQ	Mothers in intervention group reported significantly higher parental self-efficacy following intervention compared to control groups. No significant effect was found for fathers. Correlations showed that increases in maternal self-efficacy were significantly related to improvements in five mother–child outcomes	0.68 (medium)	57
^a^Tucker et al. (1998) Long term follow up of Gross et al. (1995)							Significant changes in maternal self-efficacy were maintained at 3 months and 1 year post follow up in the intervention group No significant differences were seen amongst fathers	N/A	N/A
Gross et al. (2003) USA 264	Parents: Not stated Children: 2–3 years Average age: Not stated	Inclusion: Legal guardian of 2–3 year old. Completed all baseline assessments Exclusion: Not stated	Cognitive behavioural intervention; Webster-Stratton Incredible Years BASIC program Programme topics: child-directed play, praise and rewards, effective limit setting, handling misbehaviour and problem solving. Weekly homework assignments. Teaching methods: video vignettes, group discussion. Teacher training, no intervention, teacher training and parenting group	12 × 2 h weekly sessions.	1 year follow up	TCQ	Both parent training conditions reported together against both control conditions. Growth curve modelling used to report 1 year follow up results. No significant difference post-intervention. Parental self-efficacy significantly increased in parent training groups at 1 year follow up compared to control groups Parent and teacher group showed no enhanced improvements over parent training alone	0.42 (medium)	80
Gross et al. (2009) USA 292	Parents: Not stated Children: 2–4 years Average age: not stated	Inclusion: Day centre had over 90 % of enrolled families meeting income eligibility requirements for subsidised child care. Legal guardian of 2–4 year old. Participants must speak English. Exclusion: Not stated	Cognitive behavioural intervention; Chicago Parent Program Programme topics: praise and rewards, family traditions, limit setting and consequences, stress management and problem solving skills. Teaching methods: video vignettes, group discussions, homework assignments. Waitlist control	11 × 2 h weekly sessions plus 1 booster session 2 months after 11th session	Immediate post data not reported. 1 year follow up reported	TCQ	Immediate self-efficacy results not stated. Growth curve modelling used to report 1 year follow up results. No effect of intervention on parental self-efficacy. But did find a dosage effect. Parents who attended the most sessions showed biggest improvements in self-efficacy. But no random assignment to dosage effect	Could not be calculated	78
Joachim et al. (2010) Australia 46 (96 % mothers)	Parents: 33.78 years Children: 2–6 years. Average age: 3 years	Inclusion: Parents of children 2–6 showing behaviour problems during shopping trips. Exclusion: Child had a disability and or chronic illness or if the parents were currently consulting a professional for child behaviour difficulties.	Behavioural intervention, Triple P, hassle free shopping Topics covered: engaging children in shopping, using shopping to teach appropriate behaviour and management strategies. Teaching methods: video modelling, problem solving, homework tasks and planned activity routines. Waitlist control	1 × 2 h brief discussion group	6 month follow up for intervention group only	PTC	Intervention group reported significantly higher parental self-efficacy on both the behavioural and setting scales of PTC compared to control group. The improvements were maintained at 6 month follow up. The results continued to be significant when an intention to treat analysis was completed	PTC: Behaviour subscale 1.05 (large) Setting subscale 1.25 (large)	43
Landy and Menna (2006) Canada 35	Mothers: 18–>46 years Children: 3–6 years. Average age: 4.5 years	Inclusion: Mothers of aggressive children Exclusion: Children with serious developmental delay and mothers and children with any medical condition or physical disability.	Psychodynamic intervention; HEAR program (Helping Encourage Affect Regulation) Programme topics: child development, parent’s experience of being parented, parent–child interactions, parent’s view of self and others. Teaching methods: didactic teaching, role plays, group discussion and homework tasks. Waitlist control	15 × 2 h weekly group.	No follow up	TCQ	Significant effect of intervention on parental self-efficacy, with parents who completed the intervention group reporting significantly greater parental self-efficacy than the control group	0.52 (medium)	53
Morawska et al. (2011) Australia 66 mothers 1 father	Mothers: 36.30 Father: 39.67 Children: 2–5 years Average age: 3.63 years	Inclusion: Living in Brisbane area Exclusion: Currently seeing a psychologist, do not meet age criteria	Behavioural intervention; Triple P Programme topics: reasons for disobedience, encouraging good behaviour and managing disobedience. Teaching methods: didactic teaching, video vignettes, group discussions. Waitlist control	1 × 2 h discussion group.	6 month follow up for intervention group only	PTC	Statistically significant changes in the intervention group compared to control group. Results significant when intention to treat analysis conducted. Increase in parental self-efficacy was maintained for intervention group at 6-month follow up. Control group not assessed at 6 months	PTC behaviour subscale 1.01 (large)	56
Morawska et al. (2014) Australia 86 parents	Intervention: Mothers: 35.88 Fathers: 38.06 Control: Mothers:37.00 Fathers: 39.97 Children: 2–5 years. Average age: 3.72 years	Inclusion: Parents of children aged 2–5 experiencing feeding and/or mealtime difficulties Exclusion: Receiving professional help for child, parental or marital problems. Child had disability or developmental disorder. Parent had intellectual disability or hearing impairment.	Behavioural intervention; Hassle-free mealtimes Triple P Programme Topics: psycho-education on mealtime problems, parenting traps, building routines, behavioural strategies, consistent discipline. Teaching methods: group discussion, didactic instruction, video modelling, active skills training, homework tasks Waitlist control	1 × 2 h discussion group	6 month follow up for intervention group only	CAPES	Significant increase post-intervention in both mealtime specific and general parental self-efficacy in the intervention group compared to the control group. Increase in general parental self-efficacy maintained at 6 months for intervention group	0.81 (large)	79
Wolfson et al. (1992) USA 60 families	Parents: 28.7 years Children: new-borns	Inclusion: Gestational age of at least 38 weeks. Birth weight of 5 lb or more. Apgar score of at least 6 at 5 min post birth. No gross congenital abnormalities or serious health problems. Single birth Exclusion: Not stated	Behavioural intervention; program name not stated Programme topics: infant sleep and creating good sleep habits. Teaching methods: didactic teaching, hand-outs, group discussion, daily practice diaries. Waitlist control	4 × 1–1.5 h weekly sessions: 2 pre-natal sessions and 2 post-natal sessions.	No post intervention data, Pre data compared to 4 month follow up	Adapted parental efficacy measure	Both groups showed increased parental self-efficacy from pre to post-intervention. However intervention group improved significantly more than control group. ^a^nb first rating taken at pre-birth	0.93 (large)	45
*Studies using general parental self*-*efficacy measures*									
Cunningham et al. (1995) Canada 150	Parents: Not stated Children: Range not stated Average age: 53 months	Inclusion: Child scored above 1.5 SD for parental concerns about child behaviour Exclusion: Not stated	Behavioural intervention; program name not stated Programme topics: common child management problems and parenting errors. Teaching methods: videotapes, discussion, roleplay, homework tasks. Individual treatment and wait list control	12 × 2 h weekly sessions.	6 month follow up	PSOC	Individual therapy significantly improved parental self-efficacy compared to the group intervention and waitlist control post-intervention. At 6 months, group intervention showed greatest increase from baseline; however average parental self-efficacy was equivalent across all conditions at follow up	−0.03 (no effect)	80
Hayes et al. (2008) Australia 118	Parents: 31.84 years Children: 7–40 months Average age: 8.7 months	Inclusion: Not stated Exclusion: Insufficient English skills	Behavioural intervention; program name not stated. Programme topics: settling, sleeping difficulties, breastfeeding, weaning, difficult behaviour and self-care. Teaching methods: lectures, group discussions, staff supported practice. Waitlist control	1 × 6 h intervention.	6 week follow up	PSOC	Significant increase in parent sense of competency in intervention group compared to waitlist control. This was maintained for intervention group 6 weeks post-intervention	0.74 (medium)	66
Miller-Heyl et al. (1998) USA 797	Mothers: 29.7 years Fathers: 31.5 years Children: 2–5 years Average age: 3.15 years	Inclusion: Not stated Exclusion: Not stated	Cognitive behavioural intervention; DARE to be you program Programme topics: increasing parental self-efficacy and locus of control, decision making, improving communication, stress management, psycho-education on developmental norms. Teaching methods: experiential activities, group discussions, didactic teaching, joint parent–child activities. Waitlist control	10–12 × 2.5 h weekly sessions.	1 and 2 year follow up.	SPPR	No immediate post-test results reported. At 1 and 2 year follow ups there was a significant increase in parental self-efficacy in the intervention group compared to the control group Parents who felt the least confident at the beginning benefitted the most. Changes in parental self-efficacy were related to greater nurturing child rearing. practises	Could not be calculated	42
Pisterman et al. (1992) Canada 91	Parents: Not stated Children: 3–6 years. Average age: 49 months	Inclusion: Child aged between 3–6 and not attending grade 1 school. Adequate English language ability measured by Peabody Picture Vocabulary Test Compliance to total parental commands less than 60 %. In study 2; less than 163 s on task in a standardised parent supervised task. Exclusion: Not stated	Behavioural intervention; program name not stated Study 1 focused only on compliance training, while study two included and attention span training as well. Programme topics: ADDH psycho-education, behaviour management skills. Teaching methods: role-play, modelling and homework assignments. Waitlist control	12 weekly sessions; length not stated.	3 month follow up	PSOC	Significant increase in parental self-efficacy for both intervention groups compared to the control groups. Change independent of changes in parent and child behaviour but were associated with reductions in perceived behaviour problems	0.26 (small)	45
Sheeber and Johnson (1994) USA 40	Mothers: 34 years Children: 3–5 years. Average age: not stated	Inclusion: Child showed evidence of difficult temperament–rated by parent. Exclusion: Clinical judgement if behaviour deemed due to recent stressor or psychopathology.	Temperament based intervention; name not stated. Programme topics: psycho-education on child temperament, management of temperament-related behaviour issues. Teaching methods: not stated. Waitlist control	9 × 1.5–2 h weekly sessions.	8 week follow up	PSI	Improvement in parental self-efficacy in intervention group compared to control group, maintained at follow up. Clinically significant change in parental self-efficacy for intervention group at post treatment although this was not maintained at follow up	−0.64 (medium) Reduction = improvement	38

*PTC* parenting tasks checklist, *TCQ* toddler care questionnaire, *CAPES* the child adjustment and parent efficacy scale, *PSOC* parenting sense of competency scale, *SPPR* self-perceptions of the parental role, *PSI* parenting stress index

^a^Breitenstein et al. (2012) includes dataset reported in Gross et al. (2009) alongside other data

#### Design

All but one study utilised pre-post data (Gross et al. 2009), who utilised growth curve modelling to report initial results at 12 months. In one study post-intervention data for parental self-efficacy was collected only at 4-month follow up (Wolfson et al. 1992). Subsequent follow up data were collected in 13 studies. The follow up period ranged from 6 weeks to 2 years.

#### Sample Characteristics

Sample sizes varied from 24 to 797 parents. Participants were predominantly mothers, although only two studies exclusively recruited mothers (Landy and Menna 2006; Sheeber and Johnson 1994). Three studies recruited couples (Gross et al. 1995; Pisterman et al. 1992; Wolfson et al. 1992), while the remaining studies included mixed samples consisting of the mother, father or another primary caregiver. None focused on fathers exclusively.

Children’s ages ranged from 4 months to 6 years. One study reported exclusively on parents of new-borns (Wolfson et al. 1992). Three studies reported on parents of children under 3 years of age (Gross et al. 1995, 2003; Hayes et al. 2008). Average maternal age ranged from 27 to 37 years old. Four studies did not state parental age (Cunningham et al. 1995; Gross et al. 2009; Pisterman et al. 1992; Sheeber and Johnson 1994).

### Parental Self-efficacy Measures

#### Task-Specific Measures

Ten studies used task-specific measures of parental self-efficacy. Five studies used the Toddler Care Questionnaire (TCQ; Gross and Rocissano 1988) and three studies used the Parenting Tasks Checklist (PTC; Sanders and Woolley 2001). One study used the Child Adjustment and Parent Efficacy Scale (CAPES; Morawska and Sanders 2010). The final study adapted the Parental Efficacy Measure (Bandura et al. 1980) to include items that reflected both general parenting tasks and specific tasks relating to infant sleep producing a task specific measure of parental self-efficacy.

#### General Measures

Three studies used the Parental Sense of Competency Scale [PSOC; Gibaud-Wallston and Wandersman 1978, cited in Johnston and Mash (1989)]. One study used the Sense of Competency subscale of the Parenting Stress Index (PSI; Abidin 1983), while another study used two scales from the Self Perceptions of Parental Role (SPPR; MacPhee et al. 1986).

The psychometric properties of all but two of the parental self-efficacy measures used in the reviewed studies were appraised and deemed to be psychometrically acceptable (see Črnčec et al. 2010, for a detailed review). No psychometric data on the Parental Efficacy Measure were reported (Bandura et al. 1980). The CAPES (Morawska and Sanders 2010) was found to have good internal consistency as reported in Morawska et al. (2014).

### Intervention Summary

The intervention in all the reviewed studies was parenting skills training. All bar two of the parenting skills interventions were based on behavioural or cognitive-behavioural models. The two exceptions were a temperament-based programme (Sheeber and Johnson 1994) and a programme informed by psychodynamic principles (Landy and Menna 2006). Of the 13 behavioural or cognitive-behavioural interventions, eight were based on either the Triple-P intervention programmes (Adamson et al. 2013; Joachim et al. 2010; Morawska et al. 2014, 2011) or Incredible years programmes (Breitenstein et al. 2012; Gross et al. 1995, 2003, 2009). Teaching methods across all interventions included didactic instruction, group discussions, role-plays and modelling. Many utilised video vignettes and provided homework tasks between sessions. Seven of the skills-based parenting programmes concentrated specifically on parents who reported their children had behavioural or aggressive problems (Cunningham et al. 1995; Gross et al. 1995; Hayes et al. 2008; Landy and Menna 2006; Morawska et al. 2011; Pisterman et al. 1992; Sheeber and Johnson 1994). One of these studies focussed on parents of children with an Attention Deficit Disorder with Hyperactivity (ADDH) diagnosis (Pisterman et al. 1992). The majority of interventions taught parenting skills across a range of settings; however, four interventions targeted setting-specific behaviours. Two interventions focused on feeding problems (Adamson et al. 2013; Morawska et al. 2014), one on managing shopping trips (Joachim et al. 2010) and one on getting infants to sleep (Wolfson et al. 1992). Children were present in two of the intervention groups (Hayes et al. 2008; Miller-Heyl et al. 1998).

### Length of Intervention

Intervention length varied greatly over the reviewed studies. Nine studies evaluated group intervention programmes that offered 8–15 weekly sessions, with sessions lasting between 1.5 and 2.5 h. Two studies evaluated the efficacy of interventions consisting of four 1–2 h sessions (Adamson et al. 2013; Wolfson et al. 1992). Single session group interventions ranging from 2 to 6 h were evaluated in four studies (Hayes et al. 2008; Joachim et al. 2010; Morawska et al. 2014, 2011).

### Methodological Quality

A summary of CTAM scores is presented in Table 2. Seven studies (Adamson et al. 2013; Breitenstein et al. 2012; Cunningham et al. 1995; Gross et al. 2003, 2009; Hayes et al. 2008; Morawska et al. 2014) had a CTAM score over 65, which is considered to be adequate (Tarrier and Wykes 2004).

**Table 2:** Overview of CTAM scores

Study	Sample (max 10)	Allocation (max 16)	Assessment (max 32)	Control group (max 16)	Analysis (max 15)	Active treatment (max 11)	Total score (max 100)
*Studies utilising task specific parental self*-*efficacy measure*							
Adamson et al. (2013)	**5**	**16**	**29**	**6**	**15**	**11**	**82**
Breitenstein et al. (2012)	**10**	**10**	**26**	**6**	**11**	**11**	**74**
Gross et al. (1995)	0	10	26	6	9	6	57
Gross et al. (2003)	**10**	**10**	**29**	**16**	**9**	**6**	**80**
Gross et al. (2009)	**10**	**10**	**26**	**6**	**15**	**11**	**78**
Joachim et al. (2010)	0	13	6	6	15	3	43
Landy and Menna (2006)	0	10	26	6	5	6	53
Morawska et al. (2011)	5	13	6	6	15	11	56
Morawska et al. (2014)	**5**	**16**	**26**	**6**	**15**	**11**	**79**
Wolfson et al. (1992)	7	10	3	10	9	6	35
*Studies using general parental self*-*efficacy measures*							
Cunningham et al. (1995)	**10**	**10**	**29**	**16**	**9**	**6**	**80**
Hayes et al. (2008)	**7**	**16**	**16**	**6**	**15**	**6**	**66**
Miller-Heyl et al. (1998)	5	10	6	6	9	6	42
Pisterman et al. (1992)	5	10	16	6	5	3	45
Sheeber and Johnson (1994)	0	10	6	6	5	11	38

Studies highlighted in bold scored as adequate on CTAM

#### Sample

Four studies recruited their sample from a geographic cohort; two studies utilised convenience samples and the remaining nine studies used volunteer samples. Eleven studies had a sample size greater than 27 in each intervention group but only two studies reported conducting an apriori power calculation to determine required sample size (Adamson et al. 2013; Morawska et al. 2014). Samples smaller than 27 in each group are deemed inadequate on the CTAM and no score was allocated.

#### Allocation

Every study was based on a randomised control design. Four studies were cluster randomised trials (Breitenstein et al. 2012; Gross et al. 2003, 2009; Wolfson et al. 1992), in which childcare centres or classes were randomised to condition. Five studies described the method of allocation (Adamson et al. 2013; Hayes et al. 2008; Joachim et al. 2010; Morawska et al. 2014, 2011), but only three studies stated that randomisation had been conducted independently from the research team (Adamson et al. 2013; Hayes et al. 2008; Morawska et al. 2014). Eight of studies stated that assessors were blind to treatment outcome, the exception was Hayes et al. (2008). Three studies described the method of rater blinding (Adamson et al. 2013; Cunningham et al. 1995; Gross et al. 2003), but no studies verified rater blinding.

#### Assessment

All studies used a standardised measure of parental self-efficacy with the exception of Wolfson et al. (1992), who adapted a standardised measure. Nine of the reviewed studies stated that assessments were carried out by independent assessors (Adamson et al. 2013; Breitenstein et al. 2012; Cunningham et al. 1995; Gross et al. 1995, 2009; Hayes et al. 2008; Landy and Menna 2006; Morawska et al. 2014; Pisterman et al. 1992).

#### Control Groups

Twelve of the 15 studies utilised a waitlist control or no treatment group as the only comparison group. Cunningham et al. (1995) and Gross et al. (2003) employed both a waitlist control group and at least one other intervention as comparison groups. Wolfson et al. (1992) utilised an enhanced waitlist condition to control for non-specific effects.

#### Analysis

All studies conducted appropriate analyses for their design. Seven studies conducted intention-to-treat analyses and appropriately accounted for attrition when it exceeded 15 % (Adamson et al. 2013; Breitenstein et al. 2012; Gross et al. 2009; Hayes et al. 2008; Joachim et al. 2010; Morawska et al. 2014, 2011). Five studies did not utilise an intention-to-treat analysis but did adequately account for attrition or had an attrition rate lower than 15 % (Cunningham et al. 1995; Gross et al. 1995, 2003; Miller-Heyl et al. 1998; Wolfson et al. 1992). The remaining three studies did not employ intention-to-treat analyses or made no effort to explain drop outs or adjust for differences (Landy and Menna 2006; Pisterman et al. 1992; Sheeber and Johnson 1994).

#### Active Treatment

Thirteen studies reported using a treatment protocol or manual; however, only six studies assessed adherence to the protocol (Adamson et al. 2013; Breitenstein et al. 2012; Gross et al. 2009; Morawska et al. 2014, 2011; Sheeber and Johnson 1994).

### Task-specific Measures of Parental Self-efficacy

#### Post-intervention Findings

Ten studies used task-specific measures of parental self-efficacy; nine reported parental self-efficacy findings immediately post-intervention. The exception was Gross et al. (2009) who utilised growth curve modelling to report results at 12 months post-intervention and did not specify immediate post-intervention results. Most studies reported a significant immediate intervention effect, indicating parents who completed the group intervention showed a significantly greater increase in parental self-efficacy compared to parents in control groups. Only one study did not report a significant difference post-intervention (Gross et al. 2003). The authors combined both parent training groups (parent training and parent training plus teaching training) to non-parent training conditions (teacher training and control) and found a trend for greater parental self-efficacy in their parent training groups compared to controls but this difference was not statistically significant. As means and standard deviations were reported for the four groups individually (Gross et al. 2003), a medium effect size of 0.42 was found when the parent training group was compared to the no intervention condition.

The significant effect of the intervention on parental self-efficacy was lost in the study by Adamson et al. (2013) when an intention-to-treat analysis to compensate for attrition from the study was completed. In addition, the significant results reported by Breitenstein et al. (2012) need to be interpreted with caution because baseline differences in parental self-efficacy may account for the significant result rather than any impact of the intervention. Furthermore, although a significant effect is reported, the effect size when calculated was 0 (see Table 1) and does not support the authors’ findings. Effect sizes could be calculated for all but one study (Gross et al. 2009). Medium to large effect sizes were found in eight studies; while no effect was found in the study by Breitenstein et al. (2012) (see Table 1).

#### Follow Up Assessment Findings

Eight studies reported follow up findings for parental self-efficacy (data from Gross et al. 1995 reported in Tucker et al. 1998). Four studies reported 6-month-follow up data for only the intervention group parents and found improvements in self-efficacy were maintained (Adamson et al. 2013; Joachim et al. 2010; Morawska et al. 2014, 2011). Comparisons between intervention and control groups at 6 and 12 months post-intervention were conducted in four studies (Breitenstein et al. 2012; Gross et al. 2003, 2009; Tucker et al. 1998). Equivocal findings were found: two studies reported significant improvements at follow up in parental self-efficacy in the intervention groups above the control groups (Gross et al. 2003; Tucker et al. 1998), while no differences were found in the other two studies (Breitenstein et al. 2012; Gross et al. 2009). Breitenstein et al. (2012) reported maintenance of increased parental self-efficacy in the intervention group; however after an examination of the data reported in the paper, no difference was found between control and intervention groups at any post-intervention time-point.

#### Potential Moderating and Mediating Influences

The impact of number of sessions attended was examined by Gross et al. (Gross et al. 1995, reported in Tucker et al. 1998), who found no impact of dosage on parental self-efficacy in either mothers or fathers, although the small sample size (n = 24) in this study could have reduced the power to detect an effect. Gross et al. (2009) did find a dosage effect: the intervention had a significant effect on parental self-efficacy for parents who attended at least six of the group sessions. There is evidence that attending just a single intervention session can lead to increased parental self-efficacy: significant, maintained, positive effects on parental self-efficacy were observed in the three studies evaluating single two-hour group interventions (Joachim et al. 2010; Morawska et al. 2014, 2011).

Gross et al. (1995) found different results for mothers and fathers. Mothers in the intervention group reported significantly greater maternal self-efficacy following the parenting intervention; however, no significant differences were observed in paternal self-efficacy across time or group. According to Breitenstein et al. (2012), improvements in self-efficacy also differed depending on ethnicity, with Latino parents reporting greater improvements in parental self-efficacy than African-American parents.

#### Relationship to Parent and Child Behaviour

Only one study reported on the relationship between self-efficacy and child and parent behaviour (Gross et al. 1995). Increases in mother’s self-efficacy were significantly related to reductions in child behaviour intensity, ratings of child difficult temperament and parental stress and increases in frequency of praise (Gross et al. 1995). Changes in paternal self-efficacy, although not significantly different between control and intervention groups, were associated with reductions in child behaviour problems and parental stress.

### General Measures of Parental Self-efficacy

#### Post-intervention Findings

General measures of parental self-efficacy were used in five studies. Four studies compared the intervention group and control group immediately post-intervention. Miller-Heyl et al. (1998) did not report post-intervention comparison data. The majority of studies reported a significant increase in parental self-efficacy in the intervention group compared to the control. However, the significant effect was lost when an intention-to-treat analysis was conducted in the study by Hayes et al. (2008). This was the only study using a general measure of parental self-efficacy to employ an intention-to-treat analysis. Cunningham et al. (1995) was the only study not to find a significant improvement in parental self-efficacy in parents who attended the group intervention compared to the control group. Interestingly, this study did find a significant increase in parental self-efficacy post-intervention for parents who received individual therapy compared to the group therapy and control. Effect sizes ranged from no effect (Cunningham et al. 1995) to medium (Hayes et al. 2008; Sheeber and Johnson 1994) (see Table 1).

#### Follow Up Assessment Findings

Five studies reported follow up assessment data. Length of follow up varied significantly, ranging from 6 weeks (Hayes et al. 2008) to 2 years (Miller-Heyl et al. 1998). Increased parental self-efficacy in the intervention compared to control group was maintained up to 3 months post-intervention in two studies (Pisterman, et al. 1992; Sheeber and Johnson 1994). Similar findings were reported up to 2 years post-intervention by Miller-Heyl et al. (1998). In contrast, Cunningham et al. (1995) noted that at 6 months participants in the group intervention reported greater improvements in parental self-efficacy between post-treatment and 6-month-follow up compared to those attending individual clinics. However, this result should be interpreted with caution due to a potential selective reporting bias. No comparison with the control condition is reported at follow up and further inspection of the data reported in the paper highlights that the mean self-efficacy score at follow up was equivalent across all conditions, indicating no difference.

#### Potential Moderating and Mediating Influences

Miller-Heyl et al. (1998) found their intervention had a uniform positive impact on parenting regardless of race in contrast to the findings by Breitenstein et al. (2012). Sheeber and Johnson (1994) reported that number of sessions attended did not impact on parental self-efficacy; however, they did note that attendance overall was high so this may reflect a ceiling effect.

#### Relationship to Parent and Child Behaviour

Two studies examined the relationship between parental self-efficacy and child and parent behaviour outcomes. Increases in parental self-efficacy were linearly related to greater use of appropriate limit setting and a decreased reliance on physical punishment (Miller-Heyl et al. 1998) and decreases in parental stress (Pisterman et al. 1992). However, changes in parental self-efficacy were unrelated to changes in child behaviour in the study focusing on parents of children with an ADDH diagnosis (Pisterman et al. 1992).

## Discussion

Overall the majority of the studies reviewed reported a significant positive impact of group-based interventions on parental self-efficacy in parents of children less than 6 years of age, regardless of whether a task-specific or general measure of parental self-efficacy was employed. However, the effect sizes for the interventions did appear to differ; the majority of studies which utilised task-specific measures of parental self-efficacy found medium to large post-intervention effect sizes (0.42–1.25), while small to medium effect sizes (0.26–0.74) were found in studies using general measures of parental self-efficacy.

Follow up data were reported between 6 weeks and 2 years post-intervention. Several studies only reported follow up data for the intervention group, thereby limiting the conclusions that can be drawn. Six studies included follow up data at least 6 months post-intervention for both intervention and control groups. Three studies found significant differences in parental self-efficacy at follow-up (Gross et al. 2003; Miller-Heyl et al. 1998; Tucker et al. 1998), whereas no difference was noted in the other three studies (Breitenstein et al. 2012; Cunningham et al. 1995; Gross et al. 2009). In the study by Breitenstein et al. (2012), while improvements in parental self-efficacy were maintained in the intervention group, the control group also improved over time. Furthermore, detailed, long term research is required to explore the factors underlining the long term impact of group-based interventions on parental self-efficacy.

Increases in parental self-efficacy were seen in interventions ranging from one to 15 sessions. The finding that single session interventions can significantly increase parental self-efficacy has substantial potential clinical importance in terms of the ability to disseminate time and cost effective interventions to many parents. The four studies looking at single session interventions focussed on specific problem situations, such as mealtimes and shopping. All recruited volunteer samples of parents of children without a diagnosed behavioural difficulty so greater investigation with a range of difficulties and populations would be beneficial. Group-based interventions, regardless of the length, incorporate, and draw upon, the five factors Bandura (1977, 1997) identified as important for enhancing self-efficacy; previous experience, vicarious experience, verbal persuasion, and have been found to enhance physical and psychological wellbeing (Leahy-Warren 2005; McVeigh 1998). Additional, direct comparison of brief versus longer interventions would enable an exploration of the important elements of both interventions and whether these are the same in brief and longer interventions. The long-term sustainability of the impact of single session interventions requires further investigation as none of the reviewed studies reported control group comparison data at follow up. However, the maintenance of increased parental self-efficacy in the intervention groups after one session at 6 months is encouraging. This short, time-limited approach may fit well with the tiered model of early interventions recommended by the UK Government of universal support offered to all parents before more intensive support is focussed on “at risk” families (Allen 2011; Leadsom et al. 2014).

Few studies considered if the effectiveness of group-based interventions to improve parental self-efficacy may be moderated by individual characteristics. Ethnicity was found to be a moderating factor in one study (Breitenstein et al. 2012), but not in another study (Miller-Heyl et al. 1998). Previous research has found substantial similarity in the relationship between parental self-efficacy and parental competence across ethnic groups (see Jones and Prinz 2005).

Fathers were a significantly under-reported group in the reviewed studies, with only one study reporting paternal self-efficacy (Gross et al. 1995). Yet the available research consistently highlights fathers report lower parental self-efficacy than mothers, suggesting they are an important group to target (Elek et al. 2003; Froman and Owen 1989; Hudson et al. 2001; Reece and Harkless 1998). The lack of a positive significant impact of group intervention on paternal self-efficacy compared to maternal self-efficacy found by Gross et al. (1995) implies different approaches may be necessary to increase paternal self-efficacy. Indeed, group interventions have been found to be effective in increasing paternal self-efficacy when delivered in an online forum or father-only group format (Hudson et al. 2003; McBride 1990, 1991).

Few studies directly examined the relationship between parental self-efficacy and parent and child behaviours. Thus, conclusions about whether improving parental self-efficacy is a mechanism by which group-based interventions facilitate changes in parenting competencies cannot be drawn. As such further research explicitly investigating the role of group-based parenting interventions in increasing parental knowledge and self-efficacy and how this relates to behaviour change is warranted to further build on the theory by Bandura (1977, 1982, 1997). Some support, however, was found for a correlation between increased parental self-efficacy and an increase in some positive parenting skills (Gross et al. 1995; Miller-Heyl et al. 1998). Only two studies assessed the relationship between changes in parental self-efficacy and reported child behaviour (Gross et al. 1995, Pisterman et al. 1992). While Gross et al. (1995) reported parental self-efficacy correlated with reductions in child behavioural problems, Pisterman et al. (1992) found parental self-efficacy was unrelated to changes in child behaviour in children. One possible explanation for the difference in findings may be that the children in the study by Pisterman and colleagues had greater behavioural problems as they had a diagnosis of ADDH. Further research into the mechanisms of change is needed. Does parental self-efficacy impact on child behaviour because of the impact on particular parenting behaviours, or an increase in consistency or a greater perceived ability to manage problem behaviours? How the intensity of behavioural problems mediates the relationship between parental self-efficacy and child behaviour also requires investigation. Independent assessment of child behaviours alongside parental self-report is needed to explore this.

Overall, evidence was found for a significant benefit of group interventions on parental self-efficacy. However, the methodological quality of the reported studies is important to consider. Eight out of 15 studies were rated as having inadequate quality on the CTAM. An inspection of the seven studies rated the highest quality showed that, of the six which reported post-intervention findings, four found a medium or large effect of intervention (Adamson et al. 2013; Gross et al. 2003; Hayes et al. 2008; Morawska et al. 2014) and two found no effect (Breitenstein et al. 2012; Cunningham et al. 1995). Furthermore, the effects were lost in two studies when more conservative intention-to-treat analyses were conducted (Adamson et al. 2013; Hayes et al. 2008). One possible explanation for the differing results could be differences between the sensitivity of the measures used to identify changes in parental self-efficacy as four different measures were used across the six studies. Of note, the two studies which used general measures of parental self-efficacy either found no effect or the effect was lost when an intention-to-treat analysis was conducted (Cunningham et al. 1995; Hayes, et al. 2008). As reported earlier, effect sizes were generally greater in studies utilising task specific measures of parental self-efficacy and it may be these measures are more sensitive. No clear pattern was seen in terms of sample characteristics, the content of the group or length of intervention. However, the level of description of the content of the intervention varied between papers, therefore such differences may not have been identified. Further research is clearly indicated.

Several limitations of reviewed literature were identified. Parental self-efficacy was not a main outcome measure in the majority of the studies reviewed which as noted, influences the level of information provided and the conclusions that can be drawn. Few studies also explored potential moderators. As such, further empirical testing was not possible in this review; which is noted as a limitation. Secondly, the methodology of eight out of 15 studies reviewed was judged to be inadequate. Studies with poor methodological quality often inflate the effects of psychological interventions (Wykes et al. 2008). Thus, the conclusions of the studies judged to be inadequate on the CTAM must be interpreted with caution. Thirdly, only a minority of the studies reviewed (seven studies) compared both intervention and control groups beyond the immediate post group evaluation. Additionally, the follow period was limited to 6 months post intervention in all but four studies. Greater longitudinal evaluation is required to establish any lasting impact on parental self-efficacy. Fourthly, all studies utilised self-report questionnaires as the only measure of parental self-efficacy. This is a limitation of the research area as a whole. There is not an independent measure of parental self-efficacy that can be completed by an independent rater, but this likely reflects the highly personal nature of the concept. Finally, variation exists in how the construct of parental self-efficacy is operationalised, both in terms of general versus task-specific measures and between individual questionnaires, which makes synthesis across studies more difficult. However, in a previous literature review, Jones and Prinz (2005) concluded that despite the variation in conceptualising parental self-efficacy, the different measures broadly tapped into the same concept. Nonetheless they cautioned that systematic trends in findings may be caused by different measurement strategies and this concern must be taken into account with the literature reviewed here, especially in terms of the differing effect sizes reported for general and task-specific measures. Greater consensus on the concept of parental self-efficacy and standardisation of measurement tools would aid future studies and develop this area of research.

A meta-analysis was not completed for this literature review for several reasons. While meta–analysis can be less prone to bias than narrative literature reviews (Teagarden 1989), this is only true when procedural differences between studies are low and methodological quality is consistently high. Using meta-analysis when these assumptions are violated can lead to poor, and at worst, harmful conclusions (Bailar 1995). Due to the differences between interventions (e.g. length of intervention, age range of children) and the varying quality of studies reviewed, meta-analysis was deemed inappropriate. Nevertheless, effect sizes, where possible, were calculated to aid comparison amongst studies.

The paucity of research into paternal self-efficacy highlights this is an area which requires future research. One possible area of investigation is whether different approaches are required to increase parental self-efficacy in mothers and fathers via group-based interventions. In addition, the finding that brief, single session programmes may increase parental self-efficacy warrants further investigation, including the association of this delivery format and its long-term benefits. Research so far into this format has focused on non-clinical, volunteer samples and whether similar results for single session interventions would be replicated in clinical populations remains to be seen. This review was not restricted to parenting interventions; however, no studies evaluating the impact of non-parenting group interventions on parental self-efficacy were identified in the literature search, yet there are many non-parenting group activities aimed at parents such as baby and toddler swimming, baby massage and baby and toddler music groups. These groups share many of the characteristics of parenting interventions without the explicit focus on specific parenting skills and are perhaps less stigmatising than attending a specific parenting class (Johnson et al. 2005). Their potential to improve parental self-efficacy is an area that warrants investigation. Future research also needs to examine if the mechanisms underpinning parental self-efficacy differs across child age from birth to pre-school age.
